# The Unrecognized Role of VA Call Center and Primary Care Clerical Staff in Assisting Patients with Obtaining Needed Care

**DOI:** 10.1007/s11606-021-06885-4

**Published:** 2021-06-09

**Authors:** Michael McGowan, Melissa Medich, Danielle Rose, Susan Stockdale

**Affiliations:** 1 Center for the Study of Healthcare Innovation, Implementation, & Policy, VA Greater Los Angeles Healthcare System, Los Angeles, CA USA; 2 Department of Psychiatry and Biobehavioral Sciences, UCLA Semel Institute for Neuroscience and Human Behavior, Los Angeles, CA USA

**Keywords:** primary care, access, clerical staff, veterans

## Abstract

**Background:**

VA clerks, or medical support assistants (MSAs), are a critical part of patients’ primary care (PC) experiences and are often the first points of contact between Veterans and the healthcare system. Despite the important role they might play in assisting Veterans with accessing care, research is lacking on the specific tasks they perform and what training and preparation they receive to perform their roles.

**Objective:**

Our primary aim in this study was to document MSA perceptions of their roles, the tasks they undertake helping Veterans with accessing healthcare, and additional training they may need to optimally perform their role.

**Design:**

Thematic analysis of semi-structured qualitative interviews with VA call center and PC MSAs (*n*=29) collected as part of in-person site visits from August to October 2019.

**Participants:**

MSAs at administrative call centers and primary care clinics in one large VA regional network representing 8 healthcare systems serving nearly 1.5 million Veterans.

**Key Results:**

We identified three key findings from the interviews: (1) MSAs perform tasks in addition to scheduling that help Veterans obtain needed care; (2) MSAs may not be fully prepared for their roles as first points of contact; and (3) low status and lack of recognition of the important and complex tasks performed by MSAs contribute to high turnover.

**Conclusions:**

As healthcare systems continue expanding virtual access, the roles of administrative call center and PC MSAs as first points of contact will be increasingly important for shaping patient experiences. Our research suggests that MSAs may need better training and preparation for the roles they perform assisting Veterans with accessing care, coupled with an intentional approach by healthcare systems to address MSAs’ concerns about recognition/compensation. Future research should explore the potential for enhanced MSA customer service training to improve the Veteran patient experience.

## INTRODUCTION

Improving timely access to primary care (PC) has been a longstanding goal within the Veterans Health Administration (VHA),^1^ but in spite of VA’s recent initiatives,^2^ Veteran perceptions of access have not improved as expected. Nationally, in 2019, slightly more than half (54%) of Veterans reported that they always got a helpful response as soon as they needed when they called their provider’s office.^3^ One potential explanation for the relatively low performance on Veteran perceptions of access could be that “first contact” with VA may be challenging for Veterans.

VA clerks, also called Medical Support Assistants (MSAs), are often the first people that Veterans encounter when attempting to access primary care by phone^4^ or in person. Many local VA healthcare systems have administrative call centers, staffed primarily by MSAs, that Veterans call to make appointments or leave messages for their healthcare providers (Veterans are not encouraged to call their PC providers directly). In addition, PC clinics employ MSAs to check in patients, answer phones, and perform other administrative tasks.

Previous studies of healthcare system clerks or receptionists outside VA have found that they play a key position as the entry point to the healthcare system, influencing patients’ care experiences.^5, 6, 7^ A recent VA study found that PC MSAs may function as care providers by performing a high degree of ‘emotional labor’ (e.g., handling complaints, defusing angry patients) while maintaining relatively low social status in the organizational hierarchy.^8^ Within VA, the MSA position is generally considered entry-level and one that requires little training or expertise. Although some evidence suggests that call center and PC MSAs play an important role on Patient-Centered Medical Home (PCMH) teams and influence Veterans’ perceptions of access,^9, 10^ few studies have investigated their experiences as first points of contact within call centers and PC clinics.

This project was conducted as part of an ongoing multi-year collaboration with regional and national primary care operational partners to improve Veterans’ access to primary care. Other components of the initiative included formative analysis and feedback of administrative and patient-reported access performance measures to local primary care leaders, interviews with patients about access to care,^11^ a systematic review of access management literature,^12^ and an expert panel on access management.^13^ Our approach is guided by Fortney and colleagues’ reconceptualization of access for the digital age.^14^ This framework incorporates quantifiable access measures with patients’ perceptions and expands the definition of access to include not only in-person visits, but also virtual care (telephonic, video, and asynchronous messaging).

This paper follows up on findings from our previous work on access, specifically the expert panel recommendation to prioritize patient telephone access^13^ and findings from patient interviews about attempts to make “first contact” with the healthcare system by phone and by walking into their PC clinics.^11^ We analyze qualitative data from MSA interviews to explore specific tasks they perform assisting Veterans with accessing care, the training and preparation they receive to perform their tasks, and their perceptions of their status as members of the healthcare team.

## METHODS

### Data Collection

We conducted in-person semi-structured interviews with call center (*n*=16) and PC (*n*=13) MSAs in one administrative VA region. All interviews were conducted as part of site visits to 8 administrative call centers and 8 primary care clinics between August and September 2019. The interview guide included open-ended questions about communication with patients, communication with primary care teams, quality assurance measures (e.g., audits of time to answering phones, auditing performance on calls), training offered to MSAs, and job satisfaction.

One member of the research team conducted the interview while a second member took detailed notes. We did not audio record interviews to prevent MSAs’ potential discomfort and encourage open and honest answers to interview questions. Prior qualitative studies have shown that the quality of data between transcripts and interview notes are comparable and, in some cases, analyses based on interview notes may be the better approach.^15^ To illustrate key themes in the results, we provide excerpts from the interview notes.

### Sampling and Recruitment

All MSAs were notified of the study via email explaining that participation was voluntary and that two members of the research team would be onsite for interviews. MSAs could contact the site visit team to opt-in or out of the study. We chose call center and PC MSAs from each healthcare system based on who was available on the day of the site visit, and by employment tenure (more than 1 year; less or equal to 1 year) to achieve a mix of MSAs with more or less experience. Our sample included more MSAs with longer tenure (*n*=18) than with shorter tenure (*n*=11).

### Data Analysis

Based on our previous work described above that points to the importance of Veterans’ “first contact” with the healthcare system, our interview guide was designed to elicit information about MSAs interactions with Veterans and other healthcare team members, the training and support MSAs receive to perform their work, and their experience of the work environment. Data were organized and analyzed using rapid turnaround qualitative analysis methods.^16, 17, 18^ Two members of the research team (one PhD anthropologist and one MA research assistant) each reviewed, summarized, and templated the interview summaries according to domains in the interview guide. The data reduction process of identifying dominant themes was carried out iteratively until consensus was reached. Themes were then reviewed and validated by the larger research group comprised of PhD-level health services researchers with 10+ years of experience evaluating PC initiatives. All authors attest that the activities resulting in this manuscript were not conducted as part of a research project, but as part of a non-research evaluation conducted under the authority of the Veterans Integrated Service Network (VISN) 22 Primary Care Committee.

## RESULTS

Analysis for this paper focused on MSA roles and responsibilities, training, and job satisfaction. This framing resulted in three main findings: (1) MSAs’ role assisting Veterans with obtaining needed care involves more than just scheduling; (2) MSA training may not prepare them well for assisting Veterans with obtaining needed care; and (3) low salary and lack of recognition of the important and complex tasks performed by MSAs contribute to dissatisfaction and high turnover.

### MSAs’ Role Assisting Veterans with Obtaining Needed Care Involves More than Just Scheduling

Although commonly considered to be an entry-level position requiring minimal skills, we found that call center and PC MSAs reported performing a variety of tasks related to helping Veterans access needed care (see Table 1). For example, many consider call center MSAs’ main tasks to include scheduling appointments for primary care and forwarding messages to PCPs and care teams. Veterans’ phone calls to providers often result in callback loops (many VA PC clinics do not provide telephone numbers for Veterans to call their care teams directly); Veterans phone the call center repeatedly because they either never received a call back from their PC team, or missed the call when it came. Addressing these callback loops requires call center MSAs to troubleshoot the most effective means of connecting patients with PC teams and deescalate frustrated Veteran patients.

**Table 1:** MSAs Helping Veterans Obtain Needed Care

Healthcare system challenges	Illustrative excerpts from interview notes
Problems with automated refill system	*There’s a number for patients to use an automated system (for prescriptions) but it’s not picking up their information so there’s a glitch. So frustrated patients call in for prescriptions.* *—*Notes from CC MSA interview
Medication refill questions	*Patients call and want to speak to their PCP about issues that can be handled by phone, which might be a refill, that MSA can take care of without involving the PCP.* —Notes from CC MSA interview
Veterans seeking assistance reaching their specialists	*Some patients can’t get through to specialty clinics. It’s not always a scheduling issue. MSA can give the Veteran options, but they want to talk to the actual clinic. The older Veterans want to speak to their doctors, they don’t want to send a message.* —Notes from CC MSA interview
Veteran expressing frustration at not being able to speak to their provider	*Veterans get angry when they can’t get transferred to their teams… they feel like they are getting the run around. MSA just sits there and lets them unload.* —Notes from CC MSA interview
Veteran experiencing callback loops	*The PC team might not call the patient back in a timely manner. Some clinics are notorious for that. It’s hard for MSA to take those calls. MSA reports the same people calling so knows which clinics are bad at calling back.* —Notes from CC MSA interview
Veterans cancelling and rescheduling appointments	*Rescheduling patients is challenging… that is MSA’s main problem, the constant cancellations.* *—*Notes from PC MSA interview
Veterans expressing frustration over clinic cancellations	*Providers calling in sick causes issues for patients who come a long way. MSA has figured out a lot of situations to deal with upset patients, patients in pain, and patients upset with the VA in general.* *—*Notes from PC MSA interview
Veterans experiencing problems checking in with kiosks	*There are two kiosks for patients to check in, so MSA helps patients use them. MSA works the floor. People don’t fully check in on the kiosks. When MSA is working, it’s not a problem.* *—*Notes from PC MSA interview
Veterans showing up to the wrong clinic	*There are issues where patients are in the wrong area. Patients check in for dermatology and then come to primary care, so MSA skypes the health techs to let them know.* *—*Notes from PC MSA interview
Veterans walking into PC clinic.	*MSA checks patients in and out, answers questions, relays messages, etc. Patients walk in for everything. MSA tries to get them to use the kiosks*. —Notes from PC MSA interview

*CC*, call center

Half of call center MSAs reported that their most difficult calls come from angry patients either requesting to speak directly to their providers or because they have not received a (timely) call back from their PC team. In addition, many call center MSAs reported devoting time to assisting Veterans with malfunctioning medication refill systems, allowing the patient to vent their frustration while trying to solve the problem. As a result, the call length sometimes exceeds the performance metric target of one minute or less.

*Disgruntled patients talk on and on while calls are building up in the queue. MSA did not receive training for disgruntled calls so they let patient talk and try to take notes. There’s only so much MSA can do —* Notes from CC MSA interview

Encounters with frustrated patients were not exclusive to call center MSAs. Three PC MSAs said that one of the hardest parts of coming to work was interacting with angry patients seeking resolution to healthcare system challenges. PC MSAs described tasks relating to assisting Veterans with accessing needed care by scheduling follow-up visits, showing patients how to use the check-in kiosks, communicating with other members of their PCMH team about patient needs, directing lost patients to other locations, and scheduling for non-primary care clinics (e.g., radiology, audiology).

*MSAs schedule specialty too. If the doctor puts in a consult for the eye clinic then they have to schedule. MSA has two clinics on top of their own clinic now —* Notes from PC MSA interview

In spite of operating procedures that discourage communication between call center and PC MSAs, the majority of PC MSAs reported that they received frequent, sometimes daily communication from call center MSAs, primarily via instant message or telephone. Call center MSAs contact PC MSAs with questions about scheduling appointments (e.g., appropriate length, 30 min or 60min) or to transfer patients returning phone calls they received from a member of their primary care team. As one PC MSA explained:

*PC MSAs are not supposed to answer phone calls from them (call center) but they still call primary care. MSA gets the majority of their calls because he/she answers the phone the most. States that 90% of calls are from the call center —* Notes from PC MSA interview

### MSA Training May Not Prepare Them Well for Assisting Veterans with Obtaining Needed Care

Many MSAs reported that they received inadequate training to prepare them for their role in assisting Veteran patients with obtaining needed care. MSAs described three training components: (1) New Employee Orientation (NEO), a 3-day program that introduces new employees to general VA worklife, human resources, benefits information, etc.; (2) MSA Academy, an online training that teaches MSAs to operate the multiple software programs and interfaces they will use on the job; and (3) job shadowing a more experienced MSA to learn how calls are handled in real time before taking calls without assistance. While half of the MSAs said they received each of these components, we found widespread variation in the content and length of training and some differences by type of MSA.

We found less variation in training experiences of call center MSAs as compared with PC MSAs. Two-thirds of call center MSAs reported that they completed NEO for varying lengths of time (1 day–1 week). About half said they shadowed other MSAs as part of their training, again for different lengths of time (1–2 weeks). Two call center MSAs reported that they learned mainly on-the-job with little or no formal training. In contrast, PC MSAs at all but one primary care clinic reported not receiving at least one of the three training components (NEO, MSA Academy, job shadowing).

Both types of MSAs perceived a need for more training in technical and relational skills. Some MSAs felt that they could have used more instruction on working across multiple different software platforms simultaneously to schedule patients.

*MSA says it would’ve been helpful to know how to use all the systems as they work together, rather than learn them independently. The way it’s presented is that it’s separate systems, but you need all of them open* — Notes from PC MSA interview

MSAs, particularly in call center settings, reported a need for more training on effective communication with frustrated patients or how to decompress after a difficult call. Table 2 lists additional training needs reported by both types of MSAs.

**Table 2:** Training Elements Reported as Not Provided or Inadequate to Prepare MSAs for Their Role

Training needs	Illustrative excerpts from interview notes
Reported by Call Center and PC MSAs	
On-the-job, hands-on, or in-person training	*MSA would have rather had on the job training than 3 days in the classroom.* *—*Notes from PC MSA interview
Training from people with clinical or primary care experience	*It’s important to get trained by someone from the department you’re going into. A lot of MSAs coming in get trained by someone with no PC experience.* *—*Notes from PC MSA interview
Training centered around communication and listening skills	*MSA didn’t get training on the back and forth [with patients]. It’s not mentioned at all and makes the calls longer. There should be training on communication and listening skills.* *—*Notes from CC MSA interview
Reported by PC MSAs only	
Longer duration of training	*It’s intimidating for new people. MSAs have to have their A-game when dealing with patients in person. They should extend the training.* *—*Notes from PC MSA interview
Training tailored specifically for MSA leads	*There is a lack of training for leads. They are trying to create that now, but this is an idea that they had in the past and tried to do but haven’t succeeded.* —Notes from PC MSA interview
Reported by call center MSAs only	
Training on how to handle or deescalate stressful calls	*MSA has not been trained to handle angry patients. It’s difficult to deal with these patients, hard to calm them down, hard not to raise your voice. Some MSAs hang up if Vets are too difficult or yelling at them.* *—*Notes from CC MSA interview
Training on mental health issues/mental health education	*There needs to be more training face-to-face for mental health. There are a lot of Vets with PTSD and there are a lot of scenarios, like learning how to say the right thing.* *—*Notes from CC MSA interview

*CC*, call center

### MSA Low Salary and Lack of Recognition Are Contributors to High Turnover

The MSA position is one of relatively modest salary, limited capacity for advancement, and substantial workload. These factors combined with day-to-day emotional rigors add further difficulty to an already complicated job. High turnover was reported by MSAs in both call center and primary care settings as contributing to making their jobs less desirable and/or more difficult, creating perpetually understaffed clinics and call centers:

*MSAs see a lot of people come and go. When people are fully trained, they leave. The call center feels like the training hub for the whole hospital. Nobody wants to stay in the call center —* Notes from CC MSA interview

Another call center MSA explained the link between being understaffed and its effect on patient perceptions at his/her clinic:

*The infrastructure here isn’t big enough to handle all the Vets coming in; they see it as a decline in satisfaction, but they don’t see the call center is understaffed and overwhelmed. Vets take it personally, so MSAs have to explain to them what’s actually happening —* Notes from CC MSA interview

Many MSAs expressed the desire to obtain a higher paying position, or a position with higher status and greater responsibility. When asked if they planned on being in the same position a year from now, a third of all MSAs said “no” outright. Most of those who answered “no” said they had either applied or were applying for other positions elsewhere in the healthcare system and/or wanted to “move up.” Only a handful of MSAs said definitively that they plan to work in their current role for the next year, and a few indicated that they would like to be promoted to MSA leads within that time. Another third of MSAs reported they were unsure if they would remain in the position for another year.

*Call centers are looked at as the bottom of the hierarchy. But MSAs really run med renewals and we are the hub. MSAs don’t get the paygrade and don’t get recognized enough —* Notes from CC MSA interview

## DISCUSSION

This qualitative study of Medical Support Assistants (MSAs) in administrative call centers and VA primary care clinics found the MSA role, while generally considered entry-level, is multifaceted and requires MSAs to perform a variety of tasks to help Veterans obtain care and address healthcare system challenges. Access in the digital age will increasingly incorporate virtual modes of communicating with care teams,^14^ making the MSA role as the first point of contact even more critical for shaping patient experiences. This study builds on our previous access management work pointing to the importance of Veterans’ “first contact” with the healthcare system^11, 13^ by identifying specific improvement opportunities based on the perspectives of the MSAs who patients first encounter when they walk in the door or call the VA.

The finding of MSA role complexity contributes to the literature on access to care by illustrating specific tasks that MSAs perform to assist Veterans with obtaining care. We expand on previous studies characterizing the MSA role on PCMH teams as performing “emotional labor”^8^ by describing the variety of tasks performed by call center and PC MSAs. MSAs not only answer phones, greet patients, and schedule appointments, but they also help Veterans communicate with their providers and obtain medications while bearing the brunt of Veteran frustration and complaints, both in person and on the telephone. To assist patients, MSAs in our study described “workarounds” to a standard protocol that restricts bidirectional communication between call centers and PC teams. This finding suggests that protocols discouraging bidirectional communication should be reviewed by interdisciplinary leaders and adapted as necessary to improve Veterans’ timely access to care. Future work should focus on the development of tools and protocols that facilitate bidirectional communication between call centers and PCMH teams so that PCMH teams can relay messages to call centers about how to address specific Veteran needs.

Our findings illustrate the importance of training and preparation for the MSA role, and we highlight specific types of training that MSAs may need but do not necessarily receive. While we found considerable variation in the duration and content of MSA training in both call center and primary care settings, MSAs mentioned the need for more customer service training and better preparation for navigating the multiple platforms they use simultaneously in their work. High turnover among MSAs may be in part due to the relatively low pay, low status, and high emotional labor, but could also be due to inadequate training and preparation for the job. Training deficits may compound the emotional labor MSAs experience day-to-day while reducing their ability to act as effective first points of contact^19^ between Veterans and the healthcare system. The inability of MSAs to address frustrated Veterans’ concerns and help them navigate the complex VA system could contribute to Veterans’ poor perceptions of access.

The results also confirm findings from previous research that found administrative, nonclinical employees perceived that their work was considered low status within the healthcare organization and thus undervalued.^20^ Call center and PC MSAs described several undesirable aspects of the job including minimal opportunity for professional development, high workload due to understaffing, and low pay despite the job complexity and substantial emotional labor required.^8^ Many MSAs told us they intended to leave the job within the next year. Low status and lack of recognition of job complexity likely contribute to low job satisfaction and high turnover among MSAs.

MSAs in both call center and primary care settings reported that high MSA turnover is a challenging aspect of the job. Turnover in primary care is costly,^21^ and among MSAs, it has previously been linked to negative patient perceptions of access.^22^ Given that PC MSAs are often the first employees that Veterans encounter during PC visits, their attrition could adversely impact patient experience by impeding the development of stable, lasting relationships with frontline healthcare staff.

## LIMITATIONS

Our study had some limitations. The study was conducted within one geographical VA region, and the results may not be applicable to other healthcare systems or all VAs, but may be relevant to other large, integrated healthcare systems. The MSA role and the challenges they described with helping Veterans obtain care are relevant for other healthcare organizations as struggles with PC access and care coordination are well-documented in healthcare and not unique to VA.^23, 24^

Although our sample was relatively small and split between two different settings, the frequency and consistency of the experiences reported across settings suggest we approached thematic saturation,^25^ but future research should test the validity of our findings with larger and more diverse samples. While suggestive of how call center and PC MSAs may shape Veterans’ perceptions of access, our study did not directly assess the impact of MSA job performance and training on Veterans’ access experiences. A notable strength of this study is that it is one of the very few to describe the tasks that MSAs perform to help Veterans obtain care and explore administrative call center and PC MSAs’ perceptions of their work environment, communication patterns with patients and each other, and training regimen.

## CONCLUSIONS

VA has defined primary care as a foundational service that all its healthcare facilities must provide^26^ and improving access to PC services will remain one of the organization’s top priorities in the coming years.^1^ Call center and PC MSAs are on the frontlines of this mandate and will require sufficient training and, likely, an effort on the part of the VA to address their need for more recognition and compensation in order to reduce MSA turnover.

Targeting and improving Veterans’ perceptions of primary care access may require multilevel interventions^13^ that include recognizing and valuing the important role MSAs play in helping Veterans obtain care, and standardizing MSA training to better prepare them for their role. Although our findings suggest a link between the MSA role and access, future research should investigate whether improved MSAs’ role performance is associated with better patient perceptions of access to care.
